# Does advanced maternal age explain the longer hospitalisation of mothers after childbirth?

**DOI:** 10.1371/journal.pone.0284159

**Published:** 2023-04-13

**Authors:** Anna Šťastná, Luděk Šídlo, Jiřina Kocourková, Tomáš Fait

**Affiliations:** 1 Department of Demography and Geodemography, Faculty of Science, Charles University, Prague, Czechia; 2 Department of Obstetrics and Gynaecology, Second Faculty of Medicine, Charles University, Prague, Czechia; 3 Department of Health Care Studies, College of Polytechnics Jihlava, Jihlava, Czechia; Universita degli Studi dell’Insubria, ITALY

## Abstract

**Background:**

Fertility postponement, which has comprised the most significant reproductive trend in developed countries over the last few decades, involves a number of social, personal and health consequences. The length of stay (LOS) in hospital following childbirth varies considerably between countries. Czechia, where the fertility postponement process has been particularly dynamic, has one of the longest mean LOS of the OECD member countries.

**Objective:**

We analyse the influence of the age of mothers on the LOS in hospital associated with childbirth.

**Data and methods:**

We employed anonymised individual data provided by the General Health Insurance Company of the Czech Republic on women who gave birth in 2014. Kaplan-Meier survival plots and binary logistic regression were employed to identify factors associated with long stays (> = 7 days for vaginal births, > = 9 days for CS births).

**Results:**

The impact of the maternal age on the LOS is U-shaped. A higher risk of a longer hospitalisation period for young mothers was identified for both types of birth (OR = 1.58, 95% CI 1.33–1.87, p˂0.001 for age less than 20, OR = 1.31, 95% CI 1.20–1.44, p˂0.001 for age 20–24 compared to 30–34). The risk of a longer stay in hospital increases with the increasing age of the mother (OR = 1.23, 95% CI 1.13–1.35, p˂0.001 for age 35–39, OR = 2.05, 95% CI 1.73–2.44, p˂0.001 for age 40+ compared to 30–34), especially with concern to vaginal births.

**Conclusion:**

The probability of a long LOS increases significantly after the age of 35, especially in the case of vaginal births. Thus, the fertility postponement process with the significant change in the age structure of mothers contributes to the increase in health care costs associated with post-birth hospitalisation.

## Introduction

The postponement of the fertility of women to older ages comprises the most significant reproductive trend in developed countries over the last almost three decades [1–5]. Reproductive ageing involves a number of social and personal consequences. In particular, it exerts an impact on the health of both mothers and new-born children and increases the risk of the incidence of health complications during pregnancy and childbirth [6–8].

The shift in the age at which women become mothers has also exerted an impact on medical procedures at maternity hospitals and neonatology institutions. Perhaps the most significant indicator is the steady increase in the proportion of caesarean sections performed [9, 10]. In addition, the proportion of children born following ART (assisted reproduction technique) and the associated incidence of multiple pregnancies is on the increase [11–13]. Despite this, however, the proportion of multiple pregnancies currently (2021) in Czechia does not exceed 1.2% of all births [14].

Discrepancies exist between the perceptions of obstetricians of the age of mothers and the self-esteem of mothers themselves–whereas obstetricians perceive women over the age of 35 to be high-risk mothers, the mothers may well consider themselves as still young [15, 16]. On the other hand, older mothers have been observed to be more able to accept prenatal counselling [17].

While the key factors that influence the length of hospital stay following childbirth (e.g. the type of birth, clinical risk factors, health complications, etc.) have already been investigated [18–22], the increasing age of mothers as a factor in this respect has not yet been analysed in detail.

**Fig 1:**
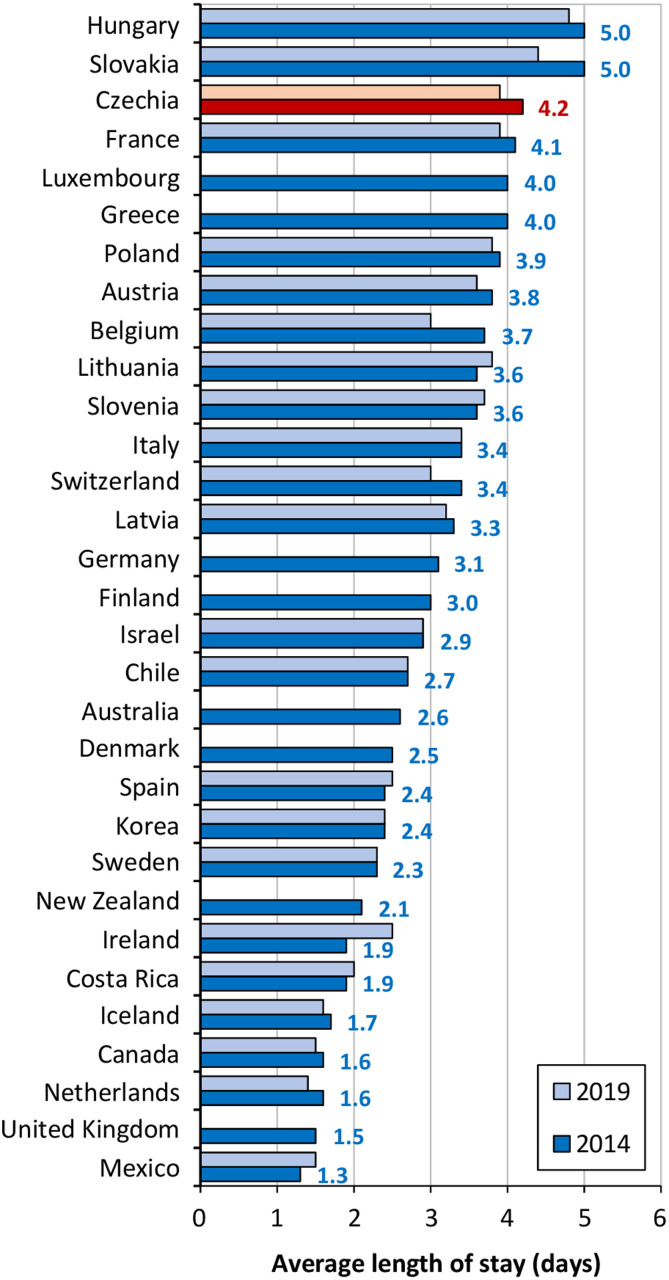
Average length of stay following a singleton spontaneous birth, selected countries, 2014 and 2019. Source: [27].

The length of hospital stay following childbirth varies considerably between countries (Fig 1). Analysis by Campbell et al. [23] showed that national norms and health system characteristics continue to influence the length of hospital stay even after adjusting for other relevant variables. Global efforts aimed at preventing maternal and perinatal mortality are targeted at ensuring that all women enjoy access to skilled childbirth attendants; the WHO recommends that all women remain in medical facilities for at least 24 hours postpartum [24] since the birth and the first 24 hours postpartum comprise the periods of highest risk for both mothers and new-borns. Thus, while in developing countries the debate focuses on the fact that the length of time that many women stay in health facilities is too short for them to receive adequate postnatal care [23], in developed countries, i.e. Western Europe, USA, Canada and Australia, on the other hand, the current trend is to shorten the relatively long period of hospitalisation following childbirth [21, 25].

Campbell et al. [23] argue that long periods of stay in health facilities following childbirth may increase the risk of exposure to the potentially adverse environment of the facility with the associated increased risk of nosocomial infections, sleep disturbance and poor infant-breastfeeding support; thus, it may act to reduce maternal confidence, paternal involvement, and family bonding, while also increasing the risk of sibling rivalry, breastfeeding problems and maternal dissatisfaction. In addition, inappropriately long periods of hospitalisation increase the financial costs for both the health system and families themselves at a time when hospitals are being encouraged to improve cost-efficiency due to a range of economic factors [26].

Czechia, along with most other Central and Eastern European countries, have the longest mean lengths of hospital stay for women following childbirth of all the OECD member countries (Fig 1) despite moves towards the shortening of the hospitalisation period in these countries in recent years. The OECD [27] reported that while in 2006 the mean length of the hospital stay following a singleton vaginal birth was 5.1 days in Czechia, in 2014 it stood at 4.2 days and at 3.9 days in 2019.

Czechia provides an example of a developed country where, although the fertility postponement process commenced in the 1990s, i.e. 20 years later than in Western and Northern Europe, its development has been particularly dynamic [28–31]. Whereas up to the early 1990s, Czechia had one of the lowest average ages of women at first childbirth (no later than 22.5 years) of any developed country, during the 1990s it witnessed one of the most rapid increases in the average age of primiparous women. The rate of growth, however, slowed down after 2000 and, in recent years, the average age at first childbirth has stagnated at 28 years, due to which Czechia does not currently feature among those countries with the oldest primiparous women. In 2016, the average age of primiparous women in Czechia stood at 28.2 years, i.e. 0.8 years lower than the EU average and almost 3 years lower than in Italy with the highest age in the EU [31].

The shift to the late fertility model is also evident from the proportion of children born to older mothers. Czechia is witnessing so-called reproductive ageing, with more and more women giving birth to their first child after the age of 30 or even 35 years [31, 32]. Whereas in 1990, women over the age of 35 accounted for only 4% of all live births, by 2019 this indicator had risen to 22% of all live births [33].

The objective of the article is to discuss, applying the example of Czechia, to what extent postpartum care in hospitals, specifically the length of hospital stay associated with childbirth, is affected by the mother’s age and to identify the extent of the involvement of other (especially health) factors that are often associated with age, namely the frequency of pregnancy, the type of birth (vaginal, caesarean section) and complications during childbirth. The analysis employed anonymised data provided by the General Health Insurance Company of the Czech Republic on the reported health care of insured women who gave birth in 2014. Since the length of hospitalisation reported by the data is measured in days of hospitalisation, and this forms the basis on which hospitals are reimbursed for the costs of the stay by health insurance companies, the article also provides important insights in terms of the debate on the financing of the health care system from public health insurance sources and the influence of the ageing of mothers as an independent factor that potentially affects the costs of the health care system.

### Data and methodology

The analysis takes into account the conditions of postpartum care in the health care system in Czechia. Perinatological care (obstetrical and neonatological) in Czechia is based on a system of differentiated, tri-level regional care provided for pregnant women and their newborns (basic–level I, intermediate–level II and intensive–level III). Intensive perinatal care (level III) is provided by a total of 12 regional perinatological centres, which focus on pre-term births up to the 32nd week. Intermediate care (level II) is provided by 15 other intermediate perinatal care centres, which focus on care for pregnant women with pre-term births up to the 34th week and pregnant women with severe cardiopathic conditions, diabetes, severe preeclampsia, intrauterine foetus growth restrictions up to the 34th week, congenital developmental defects diagnosed prenatally and other potentially life-threatening conditions for the woman or the foetus. Basic care is also provided by these centres and a further 64 level I centres with a recommended number of at least 400 births per year, taking into account the local availability of care [34, 35]. While the highly-specialised level II and III centres are located in health care facilities that serve mainly population-intensive areas (large cities), level I centres, which provide the majority of obstetric care, are spread evenly throughout the country. From this point of view, the availability of obstetric care in Czechia is at a high level, which is further reinforced by legislation that ensures the availability of inpatient gynaecological care within 60 minutes (assuming personal road transport) [36]. Czech legislation does not allow home births since midwives are not allowed to work outside hospital facilities [37].

The data employed in the analysis was taken from the General Health Insurance Company of the Czech Republic (GHIC) database [38]. The GHIC covers the majority, i.e. almost 60%, of all insured persons in Czechia [39], and has concluded contracts with the vast majority of the country’s health care facilities (it has contractual relationships with all the perinatal centres mentioned above). Moreover, the GHIC is bound by special legislation under which it is obliged to provide public health insurance if it is not provided by other health insurance companies, and to ensure the provision of affordable and high-quality health care for its policyholders [40]. Thus, the data obtained from the GHIC database can be considered to be highly representative in the context of this study since the data covers the whole of the country, as well as all the intrapartum care facilities in Czechia. At the same time, the data does not cover only a specific group of insured women (as do certain professionally-oriented or regionally-based health insurance companies), rather it encompasses the entire spectrum of the Czech population for which the insurance company legally guarantees the provision of health care.

For the purposes of the analysis, data was obtained from the GHIC database on 55,063 mothers who gave birth in 2014 at inpatient health care facilities and who were insured with the GHIC, i.e. 51.5% of the 106,971 women in total who gave birth in Czechia in 2014 [41]. The analysis employs data from 2014, which was gathered as part of the compilation of a specific set of data via which it was possible to track the health history of mothers in the years following childbirths that took place in 2014 for the purpose of further analysis. Nevertheless, as shown by Graph 1, the relevance of the data for the research question posed in this study remains valid since Czechia ranked among those countries with the longest average length of stay following a singleton spontaneous birth in both 2014 and, more recently, 2019. With respect to the analysis, it was possible to employ the database to identify both the length of the hospital stay (in days) due to childbirth and a number of the characteristics of the mothers. The data allowed for the extraction of detailed data on hospitalisation periods according to individual so-called Diagnosis Related Group (DRG) sub-databases. DRG comprises a patient classification system that assigns hospitalisation cases into the respective diagnosis group. Such data is provided at the so-called DRG base level, which corresponds to the treatment permitted to address the clinical condition as defined by the main DRG categories. Thanks to the five-digit numerical code allotted, the category can be determined by the last digit, which reflects the complexity of the case.

Subsequently, it was necessary to adjust the primary data obtained and to classify it for the requirements of the partial analysis; this led to a reduction in the overall amount of input data. The reduction reflected, in particular, the occurrence of errors in the reported care provided (e.g. the DRG code allocated to the mother was not associated with the DRG code related to childbirth). In addition, the data was removed on those mothers whose so-called hospital discharge code corresponded to either premature discharge at the woman’s request or to the death of the woman. The final data set thus contained data on 50,374 women, corresponding to 47.1% of the 106,971 women who gave birth in Czechia in 2014 [41]. The volume of primary data originally obtained was thus reduced by 8.5%. There was no information on the other half of the births since no data is available from the country’s other health insurance companies. However, we performed a detailed sensitivity analysis with respect to the differences between our study population and the total population of mothers in Czechia. The results showed that our study population does not differ in terms of the main characteristics from the total population of mothers who gave birth in Czechia in 2014.

The analysis focused particularly on the relationship between the length of hospital stay of the mother related to childbirth and the age of the woman, while controlling for other characteristics–the frequency of pregnancy, the childbirth method and complications during childbirth. Therefore, in addition to the descriptive analysis, which indicated the basic relationships between the observed variables, binary logistic regression was applied, which, together with the mother’s age as the main explanatory variable, also controlled for the influence of the other intervening variables.

The analysis considered the following variables. The **length of hospital stay** (LOS) due to childbirth served as the dependent variable. It comprises a continuous variable that indicates the length of hospital stay as the number of days that commenced in hospital. For the purposes of the regression modelling, the length of hospital stay following childbirth was dichotomised into the following categories: “normal length” (less than 7 days for a vaginal birth and less than 9 days for a caesarean section (CS) birth) and “long stay” (7 and more days for a vaginal birth and 9 and more days for a CS birth). These limits were determined on the basis of the analysis of the distribution of the length of hospital stay per type of birth and the “normal length” was set at up to the 75th percentile of the duration of hospitalisation for both vaginal and CS births (for more details see Table 1).

**Table 1:** Length of stay in hospital related to childbirth, Czechia, 2014.

Type of birth	Length of stay in hospital						N
	Mean	95% Confidence Interval for the Mean		Percentiles			
		Lower Bound	Upper Bound	25	50	75	
Vaginal	5.48	5.46	5.51	4	5	6	37,246
CS	7.51	7.43	7.59	6	6	8	13,128

Source: [38]; author’s calculations.

The main explanatory variable comprised the **maternal age at birth**, which was analysed in terms of the descriptive statistics in units of age and, for the regression analysis, was divided into six age groups: less than 20 years, 20–24, 25–29 (the reference category), 30–34, 35–39 and 40+ years. **The birth** was classified as a singleton (the reference category) or a multiple birth. **The type of birth** was divided into vaginal births (the reference category) and CS births. The vaginal birth category also included other types of delivery methods that are used only to a very limited extent (forceps 0.7%, vacuum extraction 1.8%, breech extraction 0.0% of total deliveries in Czechia in 2014 [39]). The determination of whether the birth comprised a CS or a vaginal birth was based on the reported DRG code; the CS code was 1460 and the vaginal birth codes 1461–1463. This approach to the reporting of the data meant that the vaginal births category also included the afore-mentioned minority birth types. **Complications during childbirth** (or during the hospitalisation period associated with childbirth), which are classified by hospital staff via the assignment of five-digit DRG codes into 3 basic categories–no complications (the code ends with a value of 1), complications (2) and major complications (3). These three basic categories reflect the so-called relative weighting of the case [42], which, in terms of the basic diagnosis, reflects, for example, the need to monitor the vital functions in an intensive care unit, venous access and lung ventilation, the time spent in the operating theatre and the need for the prescribing of chargeable medicines. The weighting is primarily determined from the diagnostic-therapeutic group code, which defines the upper and lower limits of the length of hospital stay and the associated material costs. For example, a spontaneous birth without complications indicates a healthy mother without any birth injuries, while a birth with complications indicates mothers with associated diagnoses (gestational diabetes, prior history of caesarean delivery, gestational hypertension etc.) or with birth injuries; a birth with serious complications covers combinations of several associated diagnoses and/or elevated costs, e.g. due to the need to treat anaemia via transfusion therapy. For the purposes of the regression modelling, this variable was dichotomised into the following categories: “without complications” (0) and “with complications” (1).

The composition of the data set in terms of the various analysed characteristics is shown in S1 Appendix.

In order to explore the distribution of the duration of hospitalisation according to the type of birth (vaginal vs. CS) and singleton or multiple birth, we employed **Kaplan-Meier curves** so as to illustrate the probability of discharge from hospital after the respective time interval. For the sake of clarity with respect to the curves, the observations were right-censored following 30 days of hospitalisation.

The **binary logistic regression** method was used in order to evaluate the extent to which the monitored variables affected the risk of the mother’s prolonged stay in hospital following childbirth. The binary logistic regression thus examined the factors associated with stays that were “long” (defined as 7 and more days for a vaginal birth and 9 and more days for a CS birth). The output of the binary logistic regression method comprises odds ratios (Exp(β) in the table), which indicate the chance of the association of a given variable category with the occurrence of “long” postpartum hospitalisation compared to the reference category. The model equation is as follows:

$$\text{logit}\left(\mathrm{Pr}\langle Y=1|x\rangle \right)=\log\left\{\frac{{}_{\mathrm{Pr}}\langle Y=1|x\rangle }{{}_{1-\mathrm{Pr}}\langle Y=1|x\rangle }\right\}=\beta_{0}+\beta_{1}x_{1}+\ldots +\beta_{k}x_{k},$$

where Y is the dependent variable (Y = 1 for a “long hospitalisation”; otherwise Y = 0), x = (x_1_, …. x_k_)’ is the vector of the explanatory variables, β_0_ is the intercept parameter and β is the vector of the slope parameters.

For the sake of clarity, the results were interpreted in terms of odds:

$$\begin{array}{c} \frac{\mathrm{Pr}\langle Y=1|x\rangle }{1-\mathrm{Pr}\langle Y=1|x\rangle }=\exp[logit(\mathrm{Pr}\langle Y=1|x\rangle )]=\exp\left(\beta_{0}+\beta_{1}x_{1}+\beta_{k}x_{k}\right)\\ =\exp\left(\beta_{0}\right)\times \exp\left(\beta_{1}x_{1}\right)\times \ldots \times \exp\left(\beta_{k}x_{k}\right), \end{array}$$

The odds ratio (exp(*β*_*k*_)) comprises a multiple by which the chances change if the value of the independent variable *x*_*k*_ changes by one and the values of the other independent variables remain unchanged.

Following the calculation of the results of the regression model, we calculated the probability of a long hospitalisation period for the defined groups of women based on the regression coefficients estimated via the model:

$$Pr(Y=1)=\frac{\exp\left(\beta_{0}+\sum \beta_{k}X_{k}\right)}{1+\exp\left(\beta_{0}+\sum \beta_{k}X_{k}\right)}$$

## Results

### Duration of hospitalisation related to childbirth

**Fig 2:**
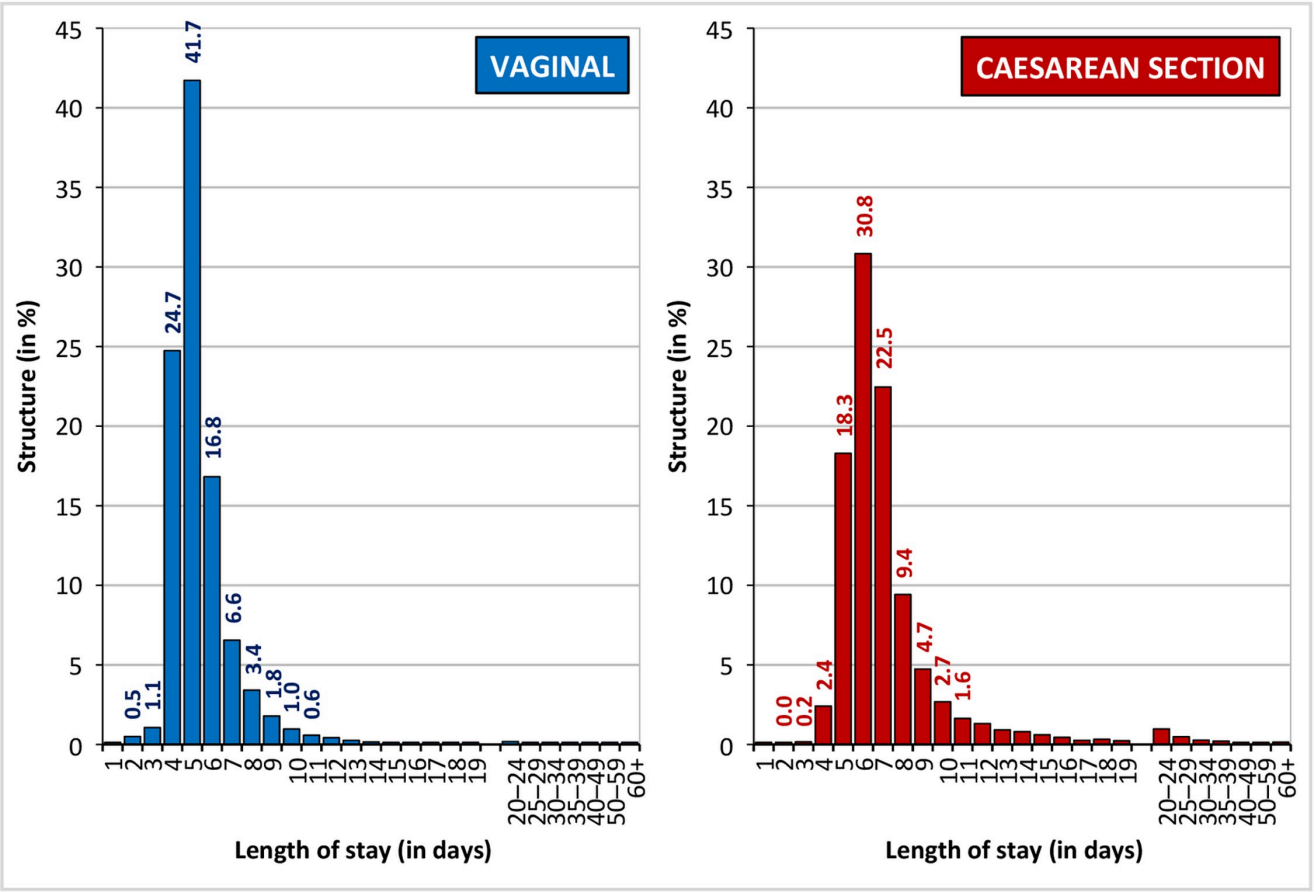
Structure of mothers according to the length of stay in hospital related to childbirth and the type of birth, Czechia, 2014, in %. Source: [38]; author’s calculations.

The most common hospitalisation period was 5 commencement days for vaginal births (42% of cases) and 6 commencement days (31% of cases) for caesarean births (Fig 2). A quarter of the women who gave birth vaginally were hospitalised for 4 days; in the case of a caesarean birth, the hospitalisation period was shorter than the modal category for every fifth mother.

The average duration of hospitalisation due to childbirth was 5.5 days for vaginal births (median 5 days) and 7.5 days for CS births (median 6 days). Table 1 illustrates that three-quarters of women were hospitalised for a maximum of 6 days following a vaginal birth and 8 days following a CS birth. We therefore applied this 75% percentile as the boundary that served to dichotomise the duration of hospitalisation variable in the logistic model and defined a “long length of stay” due to childbirth as 7 or more days for a vaginal birth and 9 or more days for a CS birth.

**Fig 3:**
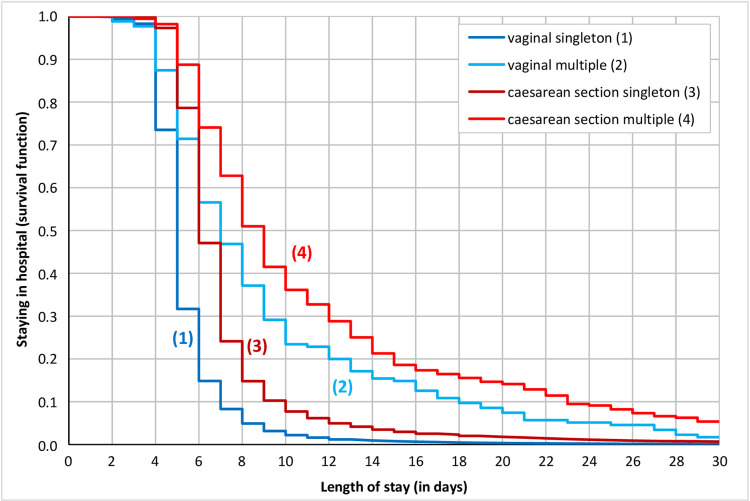
Kaplan-Meier survival curves for the length of stay in hospital, by the type of birth and singleton/multiple births, Czechia, 2014. Notes: Method: Kaplan-Meier survival plot. Dependent time variable: the length of stay in hospital related to childbirth. Source: [38]; author’s calculations.

Fig 3 shows the Kaplan-Meier survival curves for all the women according to both the type of birth (vaginal/CS) and singleton/multiple births. The graph shows that the women who leave hospital earliest are those that had a singleton vaginal birth. Moreover, in the case of singleton CS births, the duration of the hospital stay is also relatively short, i.e. mothers are discharged mostly between the 5th and 7th days of hospitalisation. Multiple births, however, serve to prolong the period of hospitalisation with respect to both vaginal and CS births.

### Duration of hospitalisation and the effect of age

**Fig 4:**
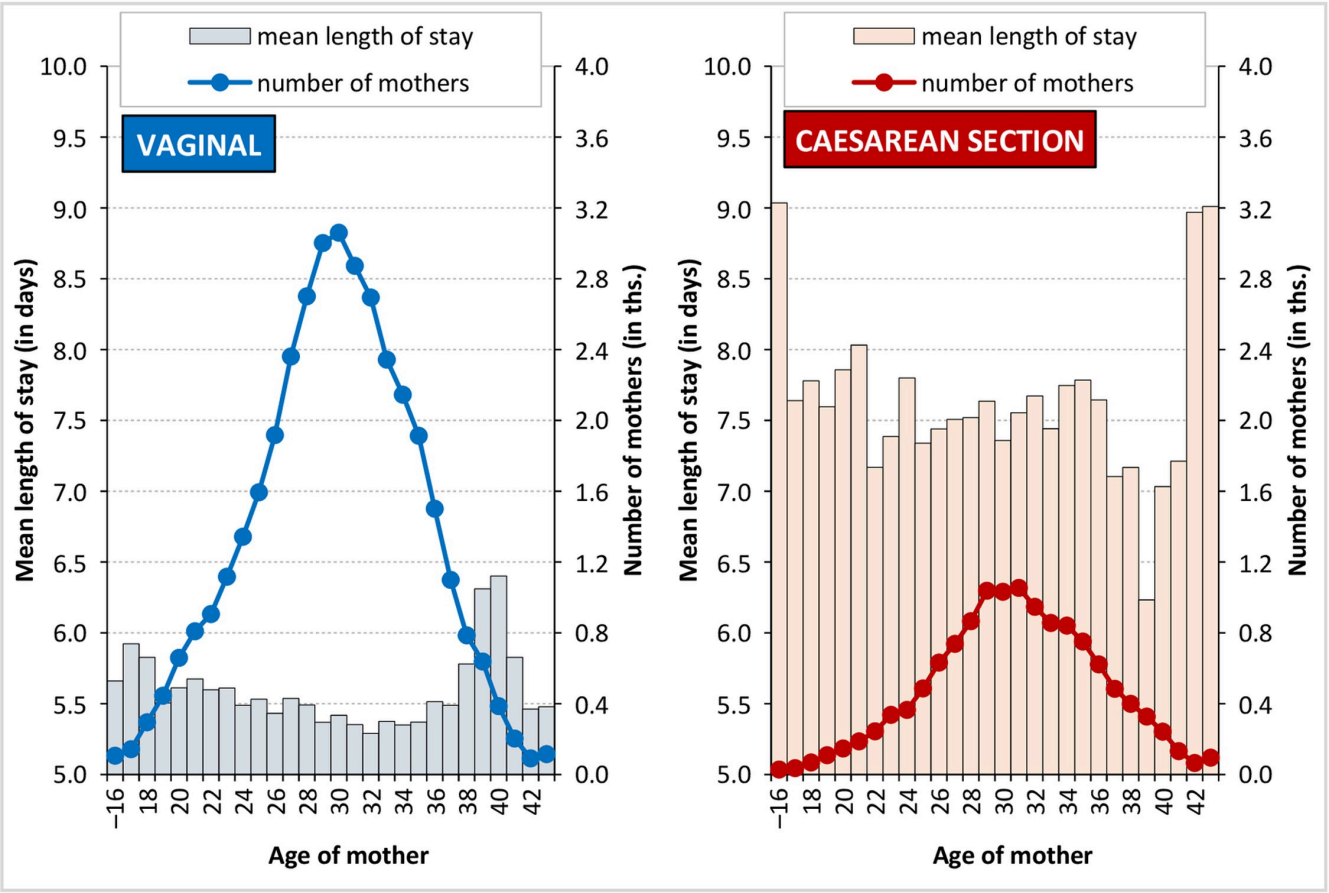
Mean length of stay in hospital following childbirth and the number of mothers by age, by type of birth, Czechia, 2014. Source: [38]; author’s calculations.

It is clear from Fig 4 that with respect to the relatively wide age spectrum of the most numerous childbirth categories (approximately from 25 to 36 years of age), the average duration of hospitalisation is shorter than for the other age groups and is relatively stable without any significant fluctuations. On the other hand, concerning the other age groups, while the average duration of hospitalisation fluctuates to a greater extent due to the lower numbers of mothers (the small numbers effect), the duration of hospitalisation is longer for both ends of the age spectrum. In the case of vaginal births, the prolongation of the duration of hospitalisation is evident after the age of 38, with an average time extension of 0.5–1.0 days more than for the most frequent childbirth age groups (S1 Appendix). Concerning CS births, the duration of hospitalisation is longer for the younger age groups as well as for the (relatively low number) highest age category of over 45 years. Conversely, a shortening of the average duration of hospitalisation by 0.5 days can be observed for the 37–42 year age group compared to the age at which most mothers give birth via CS.

**Fig 5:**
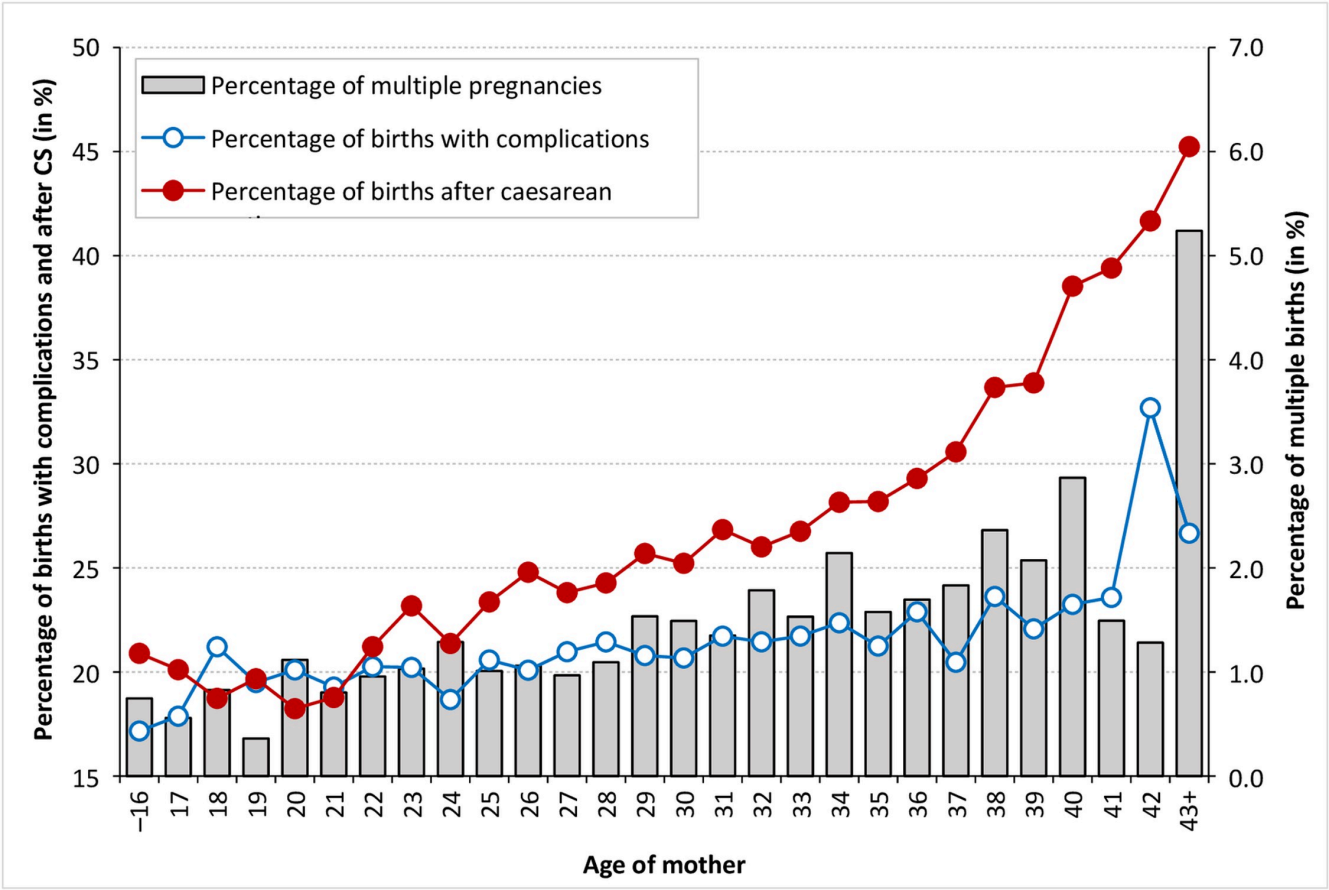
Proportion of multiple births, caesarean section births and births with complications by the mother’s age, Czechia, 2014. Source: [38]; author’s calculations.

**Fig 6:**
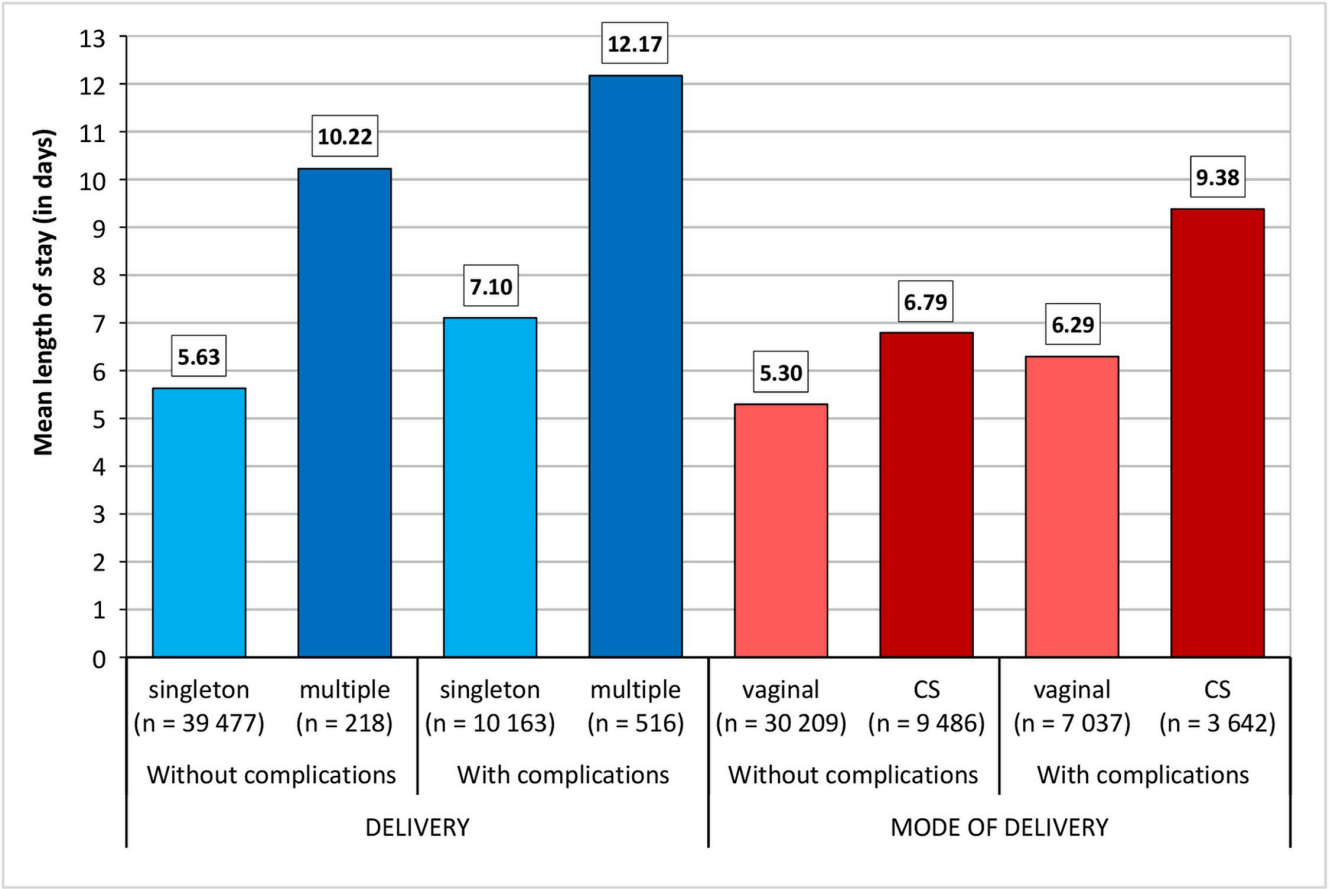
Average length of stay in hospital according to the frequency of childbirth and the type of birth depending on the occurrence of complications during childbirth, Czechia, 2014. Source: [38].

The data available to the authors allowed for the basic determination of the reasons for older mothers experiencing longer hospitalisation periods. Fig 5 illustrates that the occurrence of birth-related health complications increases significantly with the maternal age. To some extent, this may be related to the higher number of CS births and the higher proportion of multiple births experienced by older mothers, both of which are associated with longer hospitalisation periods due to health complications (Fig 6).

### The effect of age is significant especially for vaginal births

Due to the fact that the occurrence of need-related characteristics associated with the requirement for longer hospitalisation periods (multiple births, CS births, complications during childbirth) differs according to the maternal age group, a binary logistic regression model was constructed aimed at separating the influence of the mother’s age on the chance of a longer hospitalisation period following childbirth from the influence of the other factors that are closely related to the mother’s age.

Table 2 presents the results of a model into which the hospitalisation period was entered as a dependent variable in dichotomised form; the model served to estimate the chance of being hospitalised for 7 or more days in the case of a vaginal birth and 9 or more days for a CS birth.

**Table 2:** Odds ratios Exp(β) of the binary logistic models that analysed the chances of a woman experiencing a long hospitalisation period (7 days or more (vaginal birth) or 9 days and more (CS birth)) following childbirth, Czechia, 2014.

		B	OR (Exp(B))	95% C.I. for EXP(B)	
				Lower	Upper
Age of mother	–19	0.455	1.58	1.331	1.869
	20–24	0.271	1.31	1.195	1.439
	25–29	0.138	1.15	1.067	1.234
	30–34 (ref.)		1		
	35–39	0.210	1.23	1.132	1.346
	40 and more	0.719	2.05	1.725	2.443
Birth	singleton (ref.)		1		
	multiple	1.469	4.34	3.720	5.070
Complications during childbirth	no (ref.)		1		
	yes	0.965	2.63	2.489	2.769
Type of birth	vaginal (ref.)		1		
	Caesarean Section	-0.013	0.99	0.896	1.088
Interaction: age of mother x type of birth					
	19 x CS	0.182	1.20	0.841	1.712
	20–24 x CS	-0.036	0.96	0.797	1.168
	25–29 x CS	-0.064	0.94	0.814	1.080
	35–39 x CS	-0.218	0.80	0.683	0.947
	40+ x CS	-0.519	0.60	0.442	0.800
Constant		-2.123	0.120		
N		50,374			

Note: The quality of the models was verified via the application of a number of tests. The chi-square significance levels demonstrated that the included variables contributed significantly to the overall model. The Wald test and p-values for each variable category revealed which explanatory variable categories contributed significantly to the models. While the proportion of the explained variability measured via Nagelkerke R2 was just 6.3%, the model achieved a high proportion of correctly classified cases in the classification table (84.7%), thus demonstrating the good discriminatory power of the model.

The dependent variable ‘length of hospitalisation of a woman related to childbirth’– 0–6 days for vaginal and 0–8 days for CS births (0), 7 and more days for vaginal and 9 and more days for CS births (1).

Source: [38]; author’s calculations.

The results confirmed that the influence of the mother’s age on the period of hospitalisation conforms to a U-shape; the highest chance of a long hospitalisation period concerns the youngest and oldest groups of women, even when controlling for other variables that correlate with the age at childbirth. While mothers below the age of 19 have a 1.6 times higher chance of a long hospitalisation period compared to mothers aged 30–34, the chance decreases to 1.3 times for women aged 20–24 and to 1.15 times for women aged 25–29 compared to the 30–34 age group. After the age of 35, the chance of a long hospitalisation period increases to 1.23 times that of the 30–34 age group. The highest chance of a long stay in hospital concerns women older than 40 years with a twice as high chance of a long postpartum hospitalisation period than women aged 30–34 years.

Regardless of age, the type of birth and complications during childbirth increase the chance of a long hospitalisation period; multiple pregnancies increase the chance 4-fold. Complications during childbirth increase the chance of a long hospital stay 2.6 times compared to mothers who have no complications.

**Fig 7:**
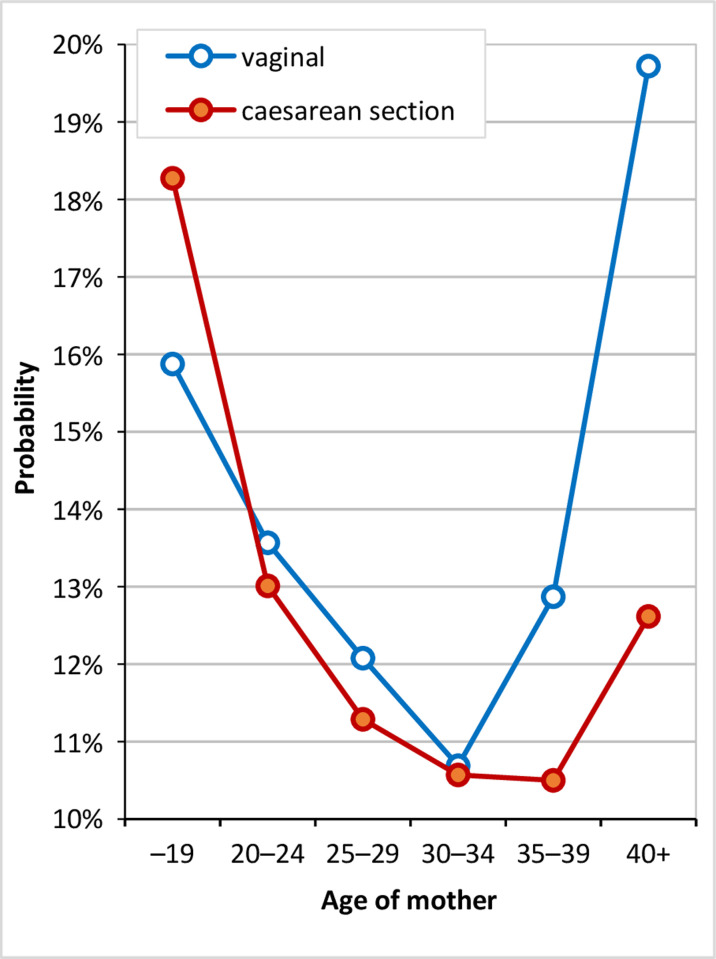
Probability of a long hospitalisation period (7 and more days for a vaginal birth and 9 and more days for a CS birth), Czechia, 2014. Source: [38]; author’s calculations.

Given the construction of the “long LOS” variable, which reflects the fact that the length of hospital stay varies depending on the type of birth, and sets a different threshold for caesarean births and vaginal births, it makes no sense to interpret the regression coefficient of the type of birth variable alone (the calculated chances do not differ since the category “long LOS” applies to approximately 15% of mothers for both types of childbirth, see S1 Appendix). However, the inclusion of the type of birth variable in the model allowed for the more detailed analysis of the effect of age depending on the type of birth; with this in mind, the interaction of these two variables was added to the model (Table 2). The descriptive analysis suggested that the length of hospital stay depending on the woman’s age may differ in the case of a vaginal birth and in that of a CS birth. This assumption was also confirmed in the logistic regression model, where this interaction was observed to be significant. In order to simplify the interpretation, the odds ratios were converted to probabilities (Fig 7). Concerning caesarean section births, the probability of a long hospitalisation period was highest for the youngest mothers and decreases towards the 35–49 age group. While for the oldest analysed age group the probability increases, the increment is only slight compared to that of vaginal births. In the case of vaginal births, very young mothers were also observed to be likely to have long hospital stays; nevertheless, we also observed a very significant increase after the age of 35. While mothers aged 30–34 have a probability of a hospitalisation period of 7 or more days in the case of a vaginal birth of only 10.5%, the probability is twice as high for women over 40 years of age.

It is important to emphasise that these results were adjusted for the impact of differences related to singleton/multiple births and the potential occurrence of complications.

## Discussion

The analysis was based on anonymised data provided by the General Health Insurance Company of the Czech Republic on health care provided to women insured by the company who gave birth in 2014. The results indicated that in 2014 a singleton vaginal birth involved an average of 5.5 days of hospitalisation and a caesarean birth an average of 7.5 days. The analysis takes into account the general conditions pertaining to postpartum care in the Czech health care system.

Our analysis was inspired by the general model of determinants of the length of hospital stay proposed by Schorr [43]. This model is based on four determinant categories: patient characteristics, clinical caregiver characteristics, characteristics of the social and family environment and the characteristics/properties of the healthcare system. With respect to the length of the postpartum hospitalisation period, Campbell et al. [23] distinguish LOS factors related to a) genuine needs (e.g. the type of birth, complications affecting the mother and/or baby) from b) the characteristics of the women, their families and communities, and from c) the characteristics of health facilities and the health care system.

In our case, the **characteristics of the Czech health care system** can be contextualised applying the recommendations of professional medical associations regarding the optimal minimum duration of hospitalisation for the mother and the child following childbirth, as well as legislative restrictions on alternatives to giving birth in health care facilities. In Czechia, the hospitalisation period is regulated by a professional recommendation that allows discharge no earlier than 12 hours after a spontaneous uncomplicated birth and on the 3rd post-operative day following a caesarean section [44]. The length of postpartum hospitalisation is also influenced by a recommendation of the Czech Neonatological Society concerning new-born children, which sets a minimum time spent in a medical facility of 72 hours prior to the discharge of a child conditional upon the child having gained weight [45]. Of the **women-related characteristics**, our primary object of interest concerned the ages of women at childbirth. With respect to reproductive ageing in Czechia, the significant change in the age structure of mothers over the last 30 years has been accompanied by a number of other age-related factors such as a significant increase in caesarean section births (from 10.3% in 1994 to 26.1% in 2014 and 23.6% in 2018 [46]), the use of assisted reproduction technologies [47] (an estimated 3.6% of mothers gave birth following ART of the total number of mothers in 2018 [10]) and an increase in the proportion of multiple births [48]. Therefore, the aim of this analysis was to evaluate the effect of the woman’s age at childbirth on the length of the postpartum hospitalisation period while **controlling for need-related characteristics** such as the type of birth (vaginal vs. caesarean), multiple births and complications during/after the birth. The aim was to form an understanding of the role of the woman’s age and the influence of other factors associated with the length of stay in hospital and the proportion of long hospital stays following childbirth.

The objective hospital length-of-stay following childbirth has decreased over the last few decades [21] despite the significant ageing of mothers, characterised by an increasing proportion of first-time mothers aged 35 and over [31]. This should be matter of concern since early discharge without home support from professional care providers is associated with the increased risk of maternal and neonatal morbidity. Moreover, older women face a higher risk of health complications related to pregnancy and childbirth than do younger mothers. We aimed to study the LOS related to childbirth and its various determinants for Czech women who gave birth in 2014. We determined significant variations in the length of stay in hospital according to the woman’s age, the type of birth (vaginal or caesarean section) and whether the woman had a singleton or multiple birth. The variation in the LOS was further attributed to complications during childbirth. Specifically, we studied the effect of age after adjusting for all the studied factors.

The financing of health care in Czechia is based on records that monitor the number of commenced days of hospitalisation, regardless of the time of admission/discharge of the woman to/from the hospital. While this approach to the reporting of the hospitalisation period provides higher incomes for health care providers (who often take advantage of the system), it results in an overall higher financial burden for the Czech health care system. The results of this study also have implications in terms of the financing of the Czech health care system. The results identified selected factors that serve to increase the likelihood of longer childbirth-associated hospitalisation stays, and which, thus, act to increase health care costs. It was revealed that not only health-related factors, which are often associated with the increasing age of mothers, but the overall older ages of mothers, especially with regard to vaginal births (and which appears to comprise an independent variable that increases the likelihood of a longer LOS [49]) contribute to increasing the costs of care covered by health insurance companies.

Our results illustrate that with respect to the Czech health care system, where professional associations recommend longer postpartum hospital stays than in most other developed countries, the LOS differs significantly according to the type of birth, singleton/multiple births and the occurrence of health complications. In 2014, the average hospitalisation period for women in Czechia who had a vaginal birth without complications was 5.3 days, and for women who had a CS birth without complications 6.8 days. Should there be complications, the LOS is extended by 1 day for vaginal births and by as much as 2.5 days for CS births.

However, in addition to the need-related characteristics, it was crucial to analyse in detail the effect of the maternal age since overall dynamic fertility postponement has resulted in significant changes in the structure of mothers. The outcomes indicated that the probability of a long LOS (defined as 7 or more commenced days for a vaginal birth and 9 or more commenced days for a CS birth) increases significantly after the age of 35 in the case of vaginal births after adjusting for the effects of multiple births and childbirth complications.

This may be associated with an age-related reduction in the probability of experiencing a stress-free puerperium [50], and increased levels of dissatisfaction with the course of the birth due to discrepancies between reality and the woman’s own perceptions [51], both of which are associated with stress [52] that works against the ready acceptance of the maternal role, including the various related responsibilities.

The probability of a long hospitalisation period following a caesarean section increases only slightly in the case of older mothers and, moreover, only over the age of 40. These results are consistent with the findings of e.g. [20], who showed that the LOS following a CS was shorter for older women (over 35 years) and women who had planned caesarean sections.

Moreover, our findings draw attention to the difference in the increase in the LOS according to the mode of delivery. The probability of a long hospitalisation stay following a CS increases only slightly for older mothers and only over the age of 40, whereas the LOS following a vaginal birth begins to increase at the age of 35. The effect of the older age of the mother in the case of a vaginal delivery is higher, which is probably due to the fact that older organisms find it harder to endure physical and mental stress and need a longer period of time to recover [53]. However, although a time of healing is required following a CS, since it is largely planned for older women, it is not associated with the acute overall exhaustion of the organism that would require significantly longer hospitalisation. Whereas a number of studies are available on the long-term consequences in cases where a surgical birth is associated with higher fatigue, no studies are available specifically covering the early postpartum period in this respect [54]. Therefore, while, in general, a spontaneous birth is more easily tolerated than a CS birth, this does not necessarily apply to older women in the early postpartum period [55].

However, we also identified the increased probability of a long hospitalisation period associated with childbirth on the opposite side of the age spectrum, i.e. concerning young mothers below 25 years of age and with respect to both types of childbirth. While in the early 1990s, most women in Czechia gave birth at this age, in recent years maximum fertility has shifted to older ages and young mothers today comprise a selective group with generally disadvantaged social backgrounds, which acts to negatively influence the course of the puerperium [50]. Young mothers in societies in which fertility is concentrated in significantly older age groups appear to face the increased risk of health complications for both the mother and the child. Kramer [56] demonstrated that the age of the mother is not a risk factor in itself, but is associated with other factors that result in, for example, the low birth weight of the child. Adolescent mothers frequently do not have a completed education, are mostly single and live in unfavourable social situations. They are also more at risk of behaviour that is detrimental to health such as smoking, alcohol and substance abuse. It is not unreasonable to assume therefore that the reflection of these factors in Czech society results in the need for longer periods of hospitalisation for the youngest age groups of mothers. Selected data on mothers [41] clearly indicates that the youngest age groups of mothers in Czechia are more likely not only to smoke during pregnancy and to commence prenatal care and ultrasound examinations at a later stage of pregnancy than older mothers, but are e.g. also more often hospitalised during pregnancy.

It is also important to mention, however, that one of the risks of shortening or of too short a period of hospitalisation lies in the probable increased incidence of the readmission of women who have a caesarean section birth. According to a study by Liu et al. [19] of readmission cases in the US, women after caesarean deliveries were more likely to be readmitted to hospital in the first week following discharge than women after vaginal deliveries. After adjusting for the maternal age, the risk of maternal readmission following a CS birth increased significantly, i.e. by 21%, 18% and 10% for mothers with hospitalisation periods of ≤2, 3 and 4 days, respectively, compared with mothers who were hospitalised for 5 days. Postpartum haemorrhage, major puerperal infection and certain hypertension disorders were associated with the elevated risk of maternal readmission and, moreover, represented the principal causes of readmission. However, an Australian study published later [25] showed that despite a decrease in the mean length of stay (see above), there was in fact a decrease in the maternal readmission rate from 3.4% in 2001 to 3.0% in 2007.

Furthermore, other studies have documented that reducing the hospital stay after a CS is becoming more prevalent and that it has not been found to be associated with adverse maternal health outcomes [57]. Programmes introduced in Denmark aimed at reducing the length of stay in hospital resulted in a nationwide decrease in the LOS following a caesarean section from 2004 to 2016 [21]. The median hospitalisation period fell by 39 hours (95% confidence interval [CI] 37.9–40.1) during this period, i.e. from 97 hours (4.0 days) in 2004 to 58 hours (2.4 days) in 2016. Reductions were observed for both planned and emergency caesarean sections. When birth- and health-related factors and demographic changes were taken into account, the median hospitalisation period fell by 30 hours (95% CI 29.3–30.8) during this period. Similarly, in Iceland, the median LOS fell significantly from 81 to 52 hours between 2007 and 2008–9, without an increase in readmissions following the introduction of fast-tract methodology for elective CS [58].

A reduction in the LOS has also been registered for complicated cases [59]. The length of hospital stay following childbirth for women with preeclampsia is associated with the risk of readmission. Wen et al. [60] determined that a longer postoperative LOS following CS birth hospitalisation was associated with a decreased risk of postpartum hypertension-related readmission. According to the adjusted analysis, postoperative LOS of 5–7 days and ˃ 7 days compared to a LOS of ˂ 3 days were associated with a decreased risk of 60-day hypertension-related readmission (adjusted risk ratios of aRR 0.59 95% CI 0.45–0.78 and aRR 0.53 95% CI 0.29–1.00, respectively). Furthermore, maternal anaemia in the third trimester serves to prolong the hospitalisation period following childbirth [61], and pre-pregnancy obesity and excess gestational weight gain (GWG) have been found to be associated with a greater risk of pregnancy complications and caesarean section [62]. Moreover, excessive GWG is associated with a longer hospitalisation period following childbirth independent of pre-pregnancy obesity, pregnancy complications and a CS birth.

It is clear from the results that the average length of hospitalisation following childbirth is still significantly longer than the recommended minimum period of hospitalisation in Czechia. The question is, therefore, whether the recommendation should be re-evaluated so as to take into account both the mode of delivery and the age of the woman.

### Limitations

The study presented herein encountered a number of limitations with respect to the data employed. The mother’s hospitalisation period may also be affected by that of the child if the s/he is unable to remain alone when receiving hospital care. The data employed did not control for neonatal factors, which may also be related to the age of the mother, e.g. neonatal immaturity and related complications [63]; an older maternal age comprises one of the risk factors for a preterm birth and the study shows that the Czech data indicates that the increasing age of mothers is related to the growth in the proportion of children born with low birth weights [64]. Undoubtedly, other variables are also related to complications following childbirth, which should ideally be controlled in the model so as to further clarify the monitored influence of the mother’s age, e.g. the childbirth order. However, the database on which the study is based has no record of such variables since, with respect to this sector, health service providers (health care facilities) are not required to submit such data to Czech health insurance companies.

## Conclusion

The increasing age of mothers exerts a number of adverse health impacts. This article focused on the impact of the increasing maternal age on the length of the maternal stay in hospital associated with childbirth. The results show that with the increasing age of the mother, the risk of a longer maternal stay in hospital due to childbirth also increases, especially in the case of vaginal births. However, the impact of the maternal age on the LOS is U-shaped, and a higher risk of a longer hospitalisation period for young mothers (up to 25 years of age) was identified with respect to both types of birth.

Prolonged hospitalisation due to the age of the mother is also associated with the increased frequency of CS births for older women. Thus, it can be assumed that the fertility postponement process contributes to the increase in the overall costs associated with post-birth hospitalisation, as previously proved by other studies [49]; a factor that is reinforced by the higher probability of longer hospitalisation periods experienced by older women, as shown in this paper.

## Supporting information

S1 Appendix Distribution of the factors (abs. and %), mean length of stay and the percentage of “long” hospitalisation cases, Czechia, 2014. Source: [38]; author’s calculations.
(DOCX)

S1 Data (XLSX)
