# Supplementary material for: Does advanced maternal age explain the longer hospitalisation of mothers after childbirth?
Source: PLoS One. 2023 Apr 13;18(4):e0284159. doi: 10.1371/journal.pone.0284159 (PMC10101530; doi:10.1371/journal.pone.0284159)
Supplement: S1 Appendix — Source: [38]; author’s calculations. (DOCX) [file pone.0284159.s001.docx]

*Appendix 1: Distribution of the factors (abs. and %), mean length of stay and the percentage of “long” hospitalisation cases, Czechia, 2014*

|  | Factor | N | % by category | Mean LOS (in days) | vaginal birth - percentage of LOS >=7 days | CS birth - percentage of LOS >=9 days |
| --- | --- | --- | --- | --- | --- | --- |
| Total | | 50,374 | 100.0% | 6.01 | 15.0% | 16.4% |
| Women-related characteristics | Age of mother at childbirth |  |  |  |  |  |
|  | –19 | 1,230 | 2.4% | 6.10 | 18.8% | 24.9% |
|  | 20–24 | 6,114 | 12.1% | 6.01 | 16.3% | 18.8% |
|  | 25–29 | 15,335 | 30.4% | 5.97 | 14.9% | 16.3% |
|  | 30–34 | 17,846 | 35.4% | 5.95 | 13.4% | 15.8% |
|  | 35–39 | 8,520 | 16.9% | 6.11 | 16.2% | 15.0% |
|  | 40–44 | 1,269 | 2.5% | 6.56 | 24.2% | 16.9% |
|  | 45+ | 60 | 0.1% | 9.12 | 18.2% | 47.4% |
| Need-related characteristics | Type of birth |  |  |  |  |  |
|  | vaginal | 37,246 | 73.9% | 5.48 | 15.0% | x |
|  | caesarean section | 13,128 | 26.1% | 7.51 | x | 16.4% |
|  | Singleton/multiple birth |  |  |  |  |  |
|  | singleton | 49,640 | 98.5% | 5.93 | 14.8% | 14.8% |
|  | multiple | 734 | 1.5% | 11.59 | 56.6% | 51.0% |
|  | Complications during childbirth |  |  |  |  |  |
|  | no | 39,695 | 78.8% | 5.65 | 12.5% | 10.8% |
|  | yes | 10,679 | 21.2% | 7.35 | 26.0% | 30.9% |

Source: [38]; author’s calculations.
